# High expression of COL5A2, a member of COL5 family, indicates the poor survival and facilitates cell migration in gastric cancer

**DOI:** 10.1042/BSR20204293

**Published:** 2021-04-09

**Authors:** Yuen Tan, Qingchuan Chen, Yao Xing, Chao Zhang, Siwei Pan, Wen An, Huimian Xu

**Affiliations:** 1 Department of Surgical Oncology, First Affiliated Hospital of China Medical University, Shenyang, China; 2 Department of Cell Biology, Key Laboratory of Cell Biology of Ministry of Public Health, and Key Laboratory of Medical Cell Biology of Ministry of Education, China Medical University, No. 77, Puhe Road, Shenyang North New Area, 110122 Shenyang, Liaoning, China

**Keywords:** advanced gastric cancer (AGC), COL5 family, COL5A2, TCGA and GEO databases, WGCNA

## Abstract

Background: Gastric cancer (GC) metastasis determines the prognosis of patients, and exploring the molecular mechanism of GC metastasis is expected to provide a theoretical basis for clinical treatment. Recent studies have shown that extracellular matrix protein is closely related to GC metastasis. The present study aimed to explore the expression profile and role of COL5A2, as an extracellular matrix protein, in GC.

Methods: The expression, overall survival, and progression-free survival data of COL5 family members were extracted from The Cancer Genome Atlas (TCGA) database, respectively. Weighted gene co-expression network analysis of the GSE62229 database was performed out to identify modules and associated genes.

Results: COL5A2 was selected as our research target in the TCGA database, and was also verified in the GSE62229 and GSE15459 datasets. COL5A2 was up-regulated in GC tissues by paraffin immunohistochemistry and RT-qPCR. The prognosis of patients with low COL5A2 expression was better than that of patients with high COL5A2 expression. Scratch and migration experiments showed that knockdown of COL5A2 decreased the migration ability of gastric cancer cells compared with the control group. *In vivo*, mice with tail vein injection COL5A2 knockdown had fewer and smaller metastatic nodules in liver. GSEA results showed that the TCGA and GSE62229 samples were significantly enriched in several well-known cancer-related pathways, such as the TGF-β, MAPK, and JAK2 signaling pathways.

Conclusion: COL5A2 was most closely related to advanced GC among COL5 family members. High COL5A2 expression is associated with a poor prognosis, and may be a novel therapeutic target for GC.

## Introduction

Gastric cancer (GC) is a common malignant tumor of the digestive tract, and the global incidence and mortality of GC are ranked fifth and second, respectively [1,2]. Presently, the survival of GC patients has been significantly prolonged by the combination of radical surgery with radiotherapy and chemotherapy; however, the prognosis of advanced or metastatic patients remains unsatisfactory [3,4]. Because the symptoms of early GC lack specificity, most of the patients are diagnosed in the middle and late stages. Thus, identifying abnormally expressed genes in GC and intervening are important strategies to prolong the survival time of GC patients.

Collagen is the main component of the extracellular matrix (ECM), which can be divided into types I–V [5]. Type V collagen (COL5), an important component of the ECM, can regulate the diameter of fibers by interacting with type I collagen during fiber development [6]. The COL5 family comprises three main isomers, with three different polypeptide α chains, A1, A2, and A3. The abnormal expression of the COL5 family in tumors affects malignancy and progression, but the clinical role and molecular mechanism of the COL5 family in GC remain unclear [7–9].

Previously, high-throughput bioinformatics approaches, such as gene chip and gene sequencing, have been widely used to identify cancer biomarkers [10]. Some high-throughput storage databases are publicly available [11,12], and investigators can reuse these databases for data mining according to their study design. Gene co-expression network analysis (WGCNA) is a powerful biology method to analyze the correlation patterns among genes in RNA-seq or microarray samples [13,14]. The method clusters highly correlated genes into the same module and connects them with clinical traits, which may be more conducive to the identification of clinical biomarkers for diagnosis and treatment. This method has been generally recognized in cancer research and has successfully identified targeting modules and hub genes [15,16].

In the present study, we performed The Cancer Genome Atlas (TCGA) analysis on expression, overall survival (OS), and progression-free survival (PFS) microarray data to identify the COL5A family gene that is significantly associated with GC metastasis. Moreover, we explored the related genes and predicted the pathway through WGCNA analysis of GSE62229 database.

## Materials and methods

### Cell culture

We purchased three GC cell lines (SGC-7901, MGC-803, and HGC-27) and one immortalized human gastric epithelial mucosa cell line (GES-1) from the Cell Culture Collection of the Chinese Academy of Sciences (Shanghai, China). The cells were cultured in DMEM medium supplemented with 10% fetal bovine serum (FBS; Biological Industries, Israel).

### Data sources and data preprocessing

The TCGA Stomach Adenocarcinoma (STAD) data set contains 408 cancer cases and 211 matched paracancerous tissues. We used GEPIA (http://gepia.cancer-pku.cn/) to compare the gene expression differences, OS and PFS of COL5A1, COL5A2 and COL5A3 in TCGA, so as to select the most significant different expression genes (DEGs) of COL5 family.

The pretreated expression profiles of the GSE62229 and GSE15459 datasets, with high quality and quantity of GC cases, were downloaded from the GEO database. The OS and PFS of the two patient databases were detected using the K-M plotter [17]. GSE62229 is a microarray dataset containing 300 cancer tissue samples and 100 cases of paracancerous tissues, and its clinical characteristics are very complete. This dataset was selected as the training data for further study.

### Samples and patients

We used 48 pairs of fresh specimens and adjacent non-cancerous tissues from the First Affiliated Hospital of China Medical University in 2018. We also used 126 paraffin-embedded GC tissues and 60 adjacent normal tissues from patients treated between 2011 and 2012. All the patients were confirmed to have gastric adenocarcinoma pathologically, no tumor was found in other regions, and no radiotherapy or chemotherapy was performed before the operation. The patients or their families sign informed consent. The present study was approved by the research ethics committee of our institute.

### SiRNA knockdown and overexpression plasmid

Silenced COL5A2 cells can be obtained by transfection siRNA (Origene, China). The sequence was AGAAGUCACACUAGUAUAUACCATT. The expression levels of COL5A2 could be tested by RT-qPCR. The overexpression plasmid of COL5A2 was purchased from the company of Beijing Syngentech. HGC-27 stable transfer cells could be screened by G418 (Dingguo,Beijing).

### Wound-healing assay

The cells digested with pancreatin were seeded in six-well plates with the cell density for 1 × 10^5^ cells/ml. When the cell confluence reached 80–100%, the cells were scratched with 100 µl pipette tip. PBS was used to wash off the floating cells. About 2 ml FBS-free medium was added, and then the plates were photographed under a microscope (100× magnification). The plates were placed in an incubator at 37°C for 24 h, and the scratch was again observed and photographed.

### Migration assay

The cells were digested and resuspended in serum-free medium, and then 200 µl of cell suspension at 3 × 10^4^ cells/ml was seeded in Transwell chambers (8 μm pore size; Corning, U.S.A.). We then added 600 µl of medium containing 10% FBS to 24-well plates. The cells were fixed and stained after 20 h in an incubator at 37°C, and the migrated cells were counted under a microscope, which was considered to represent the migration ability.

### Screening of DEGs

The R software based on the ‘Limma’ R package was used to screen the DEGs between GC tissue and adjacent normal tissue for GSE62229. A false discovery rate (FDR) <0.05 and |log 2 (FC)| ≥0.263 were regarded as the cut-off thresholds.

### Construction of the Co-expression network

After determining the DEGs’ expression data from the GSE62229 dataset, a co-expression network was conducted for downstream analysis using the ‘WGCNA’ R package. WGCNA could effectively combine gene expression information with the clinicopathological features to identify potential modules. Next, Gene Ontology (GO) and Kyoto Encyclopedia of Genes and Genomes (KEEG) enrichment analyses were used to assess the functional role of the module genes based on R software [18,19].

### Gene set enrichment analysis (GSEA)

To determine the possible pathway through which COL5A2 functions in the development of GC, the expression data from GSE62229 and TCGA were also used to perform Gene Set Enrichment Analysis (GSEA) [20,21]. According to the differences in expression, the database cases were uniformly divided into low-expression and high-expression groups.

### Real-time quantitative PCR (RT-qPCR) analysis

The tissues were cut and homogenized. After extracting the total RNA according to the instructions, cDNA templates were generated by reverse transcription by PrimeScript™ RT Kit (TaKaRa, Japan). Real-time polymerase chain reaction was performed to calculate relative expressions of mRNA according to the reaction system. The number of cycles was set to 40. GAPDH was chosen as the reference gene. The primer sequences of COL5A2 were 5′-CAGGCTCCATAGGAATCAGAGG-3′ (sense) and 5′-CCAGCATTTCCTGCTTC TCCAG-3′ (antisense).

### Immunohistochemistry

Immunohistochemistry (IHC) staining was performed according to standard protocols. IHC staining was assessed by scores based on the percentage of positive cells (0: <5%; 1: 5%–25%; 2: 25%–50%; 3: 50%–75%; 4: >75%) multiplied by scores based on the intensity of staining, (0: colorless; 1: light yellow; 2: brown; 3: dark brown), with 6–12 considered high expression and 0–4 considered low expression. The primary antibody against COL5A2 used in IHC testing was purchased from LifeSpan BioSciences, lnc (Seattle, WA, U.S.A.).

### Western blot

The steps of Western blot followed our previous article [22]. TGF-β antibody was purchased from Proteintech (Wuhan, China), and flag antibody was from ABclonal (Wuhan, China).

### Liver metastasis assay

For the xenograft model, 2 × 10^6^ SGC-7901 cells were injected into the tail vein of 5-week-old female Balb/c nude mice, which were randomly divided into two groups with five mice in each group. After a week, the experimental group injected 1 nmol siCOL5A2 with twice a week, and the control group only received an injection of same amount of saline. A total of 2 × 10^6^ Control / COL5A2 OE HGC-27 cells were also divided into two groups and injected into tail vein of nude mice. Three weeks after the injection, all the nude mice were anesthetized with 40 mg/kg pentobarbital sodium and were put to death by cervical dislocation. The metastasis was determined by observing the disseminating degree and size of the tumor in the liver. All animal experiments were approved by the Animal Ethics Committee of China Medical University.

### Statistical analysis

Statistical analysis was performed using SPSS 22.0 statistical software and GraphPad Prism7.0 mapping software. Student’s *t*-test was used to compare the two groups. The Kaplan–Meier method was used to calculate OS. *P*<0.05 was considered statistically significant.

## Results

### COL5A2 is up-regulated in GC tissues and correlates with poor survival in the TCGA and GEO databases

**Figure 1:**
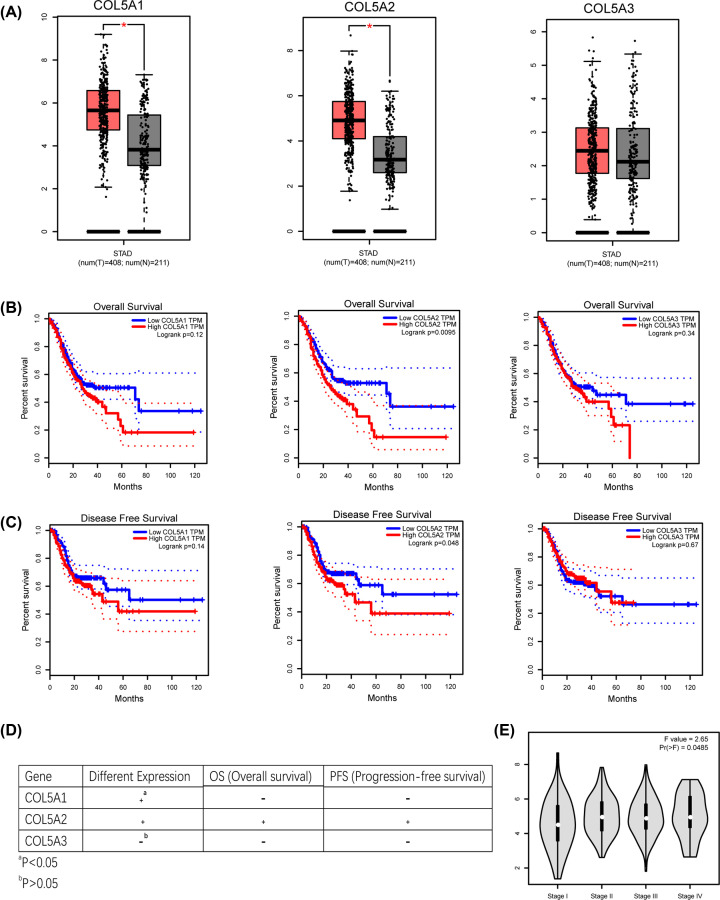
Expression and survival analysis of the COL5 family in the TCGA-STAD cohort (**A**) Box plot of the expression levels of the three genes in GC from the TCGA database. The *x*-axis shows the number of GC samples and normal samples, and the *y*-axis shows the gene expression levels. The *P* value was determined using Student’s *t*-test, and error bars were represented as means ± s.d. (**B** and **C**) Kaplan–Meier survival curves of the OS and PFS for three genes were plotted. The *P* value was determined using the log-rank test. (**D**) COL5A2 was found to be statistically significant in both expression and survival in patient samples, compared with COL5A1 and COL5A3. (**E**) Relationship between COL5A2 and T stage based on the TCGA-STAD data. *P*<0.05 represents statistical significance. *P<0.05

First, TCGA-STAD was used to predict the mRNA expression levels of three major isomers of the COL5 family in GC and adjacent normal tissues. COL5A1 and COL5A2 were up-regulated in GC compared with COL5A3 (*P*<0.05) (Figure 1A). To evaluate the prognostic value of the COL5 family mRNA expression in GC, Kaplan–Meier analysis and the log-rank test were used to verify the relationship between mRNA expression and OS or PFS in GC patients. In patients with high COL5A2 expression, OS and PFS were significantly reduced (*P*<0.05); however, COL5A1 was only have a significant trend in OS (*P*=0.12) and PFS (*P*=0.14) (Figure 1B,C). Analysis of T stage showed that COL5A2 expression in advanced GC was significantly higher than that in early GC (Figure 1E). The above analysis showed that high COL5A2 expression indicated a poor prognosis of GC. Therefore, we chose COL5A2 for further exploration (Figure 1D).

**Figure 2:**
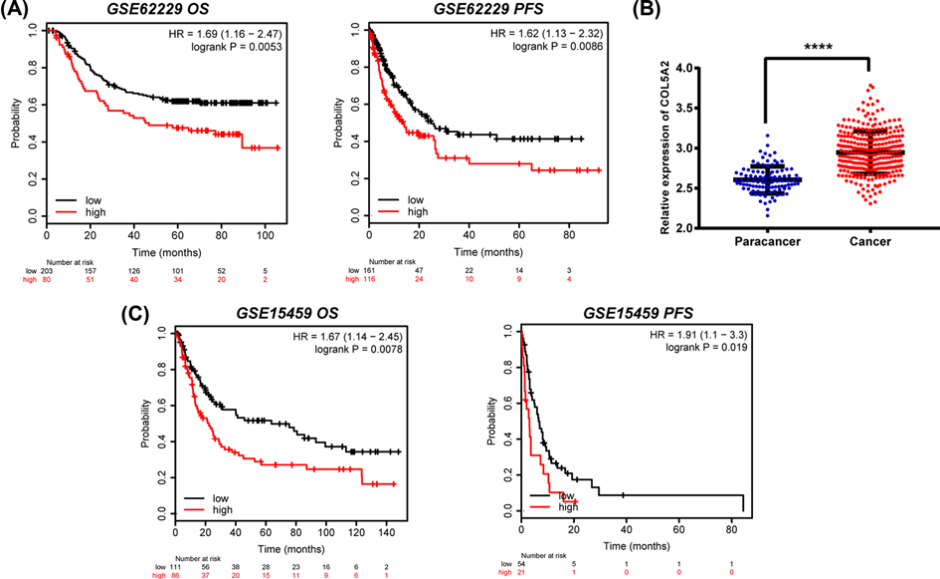
COL5A2 is up-regulated in GC tissues and correlates with poor survival (**A** and **C**) The K-M plotter shows the survival curves of COL5A2 in GSE62229 and GSE15459. Overall survival = OS, and progression-free survival = PFS. *P*<0.05 represents statistical significance. (**B**) The point map shows the COL5A2 expression level in GC tissue (*n*=200) and paracancerous tissue (*n*=100) from GSE62229. ****P<0.001. *P*<0.05 represents statistical significance.

To verify the findings in the TCGA database, the GSE62229 and GSE15459 datasets were selected to evaluate the expression and prognosis of COL5A2. COL5A2 expression in cancer tissue was significantly higher than that in adjacent normal tissues (*P*<0.001) (Figure 2B). Additionally, in the two GEO databases, patients with low COL5A2 expression showed longer OS and PFS (Figure 2A,C).

### High COL5A2 expression indicates a poor prognosis in GC tissues

**Figure 3:**
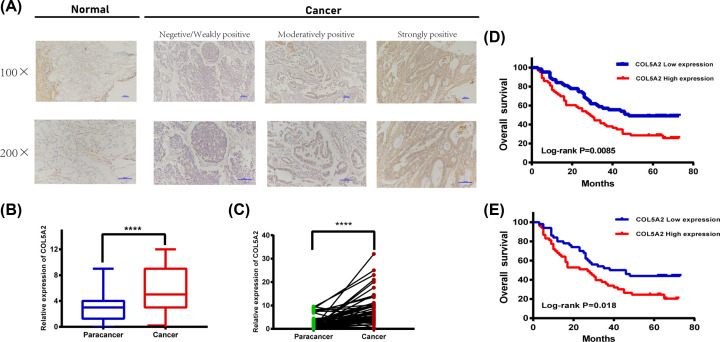
Detection of COL5A2 protein and mRNA expression in patient tissues (**A**) Representative images of COL5A2 protein expression in GC and normal tissues. IHC scoring based on the staining intensity was performed. The scale bar is displayed. (**B**) COL5A2 protein expression was significantly increased in primary tumor tissues (*n*=126), compared with that in adjacent normal tissues (*n*=60) by IHC. (**C**) RT-qPCR detected the COL5A2 mRNA level of the 48 paired fresh specimens and adjacent non-tumor tissues. (**D**) Kaplan–Meier analysis of COL5A2 in GC patients. Patients with low COL5A2 expression had longer OS than those with high COL5A2 expression (*P*=0.0085). (**E**) Kaplan–Meier analysis of COL5A2 in advanced GC patients (*P*=0.018). ****P<0.001. *P*<0.05 represents statistical significance.

To validate the possible role of COL5A2 in GC progression, the expression pattern of COL5A2 was explored in paired clinical tissue samples in our patient samples. Thus, 126 paraffin-embedded GC tissues and 60 adjacent normal tissues with complete clinicopathological variable and follow-up information were collected. The COL5A2 protein level was significantly higher in GC tissues than in normal tissues (*P*<0.001; Figure 3A,B). Next, we used RT-qPCR to assess the expression pattern of COL5A2 in 48 pairs of fresh specimens and adjacent non-cancerous tissues (Figure 3C); the findings were consistent with the IHC results. Taken together, these results confirmed that COL5A2 is highly expressed in GC tissues.

Next, the prognostic role of COL5A2 was confirmed in our samples. Based on the COL5A2 expression levels, patients with complete follow-up information were divided into the COL5A2 low-expression group (negative or weakly positive expression, *n*=64) and COL5A2 high expression group (moderately or strongly positive expression, *n*=64). Kaplan–Meier curves confirmed that patients with high COL5A2 expression had a significantly shorter OS than those with low COL5A2 expression (*P*=0.0085, Figure 3D). Additionally, we verified the significance of COL5A2 in the survival of advanced GC (*P*=0.018; Figure 3E).

The association between COL5A2 expression and clinicopathological parameters in patients with GC was further evaluated. As shown in Table 1, COL5A2 expression in GC was correlated with Borrmann type (*P*=0.036), histological type (*P*=0.013), and T stage (*P*<0.011). A significant correlation was not found between COL5A2 and age, sex, tumor location, tumor size, or N stage. These results confirmed that COL5A2 expression is associated with the malignant phenotype of GC.

**Table 1:** Correlation between COL5A2 expression and clinicopathologic features in GC patients

Factor	COL5A2 expression		*P* value
	Low (*n*=63)	High (*n*=63)	
Sex			0.716
Male	39	37	
Female	24	26	
Age			0.373
<60	30	35	
≥60	33	28	
Tumor location			0.477
Upper	8	13	
Middle	12	10	
Lower	43	40	
Tumor size			0.229
<4cm	20	14	
≥4cm	43	49	
Borrmann type			**0.036**
I-III	53	43	
IV	10	20	
Histological type			
Well/moderate	38	24	**0.013**
Poor	25	39	
T stage			**0.011**
T1-3	33	19	
T4	30	44	
N stage			0.193
N0-2	44	37	
N3	19	26	

### Silenced COL5A2 inhibited the migration of GC cells *in vitro* and *in vivo*

**Figure 4:**
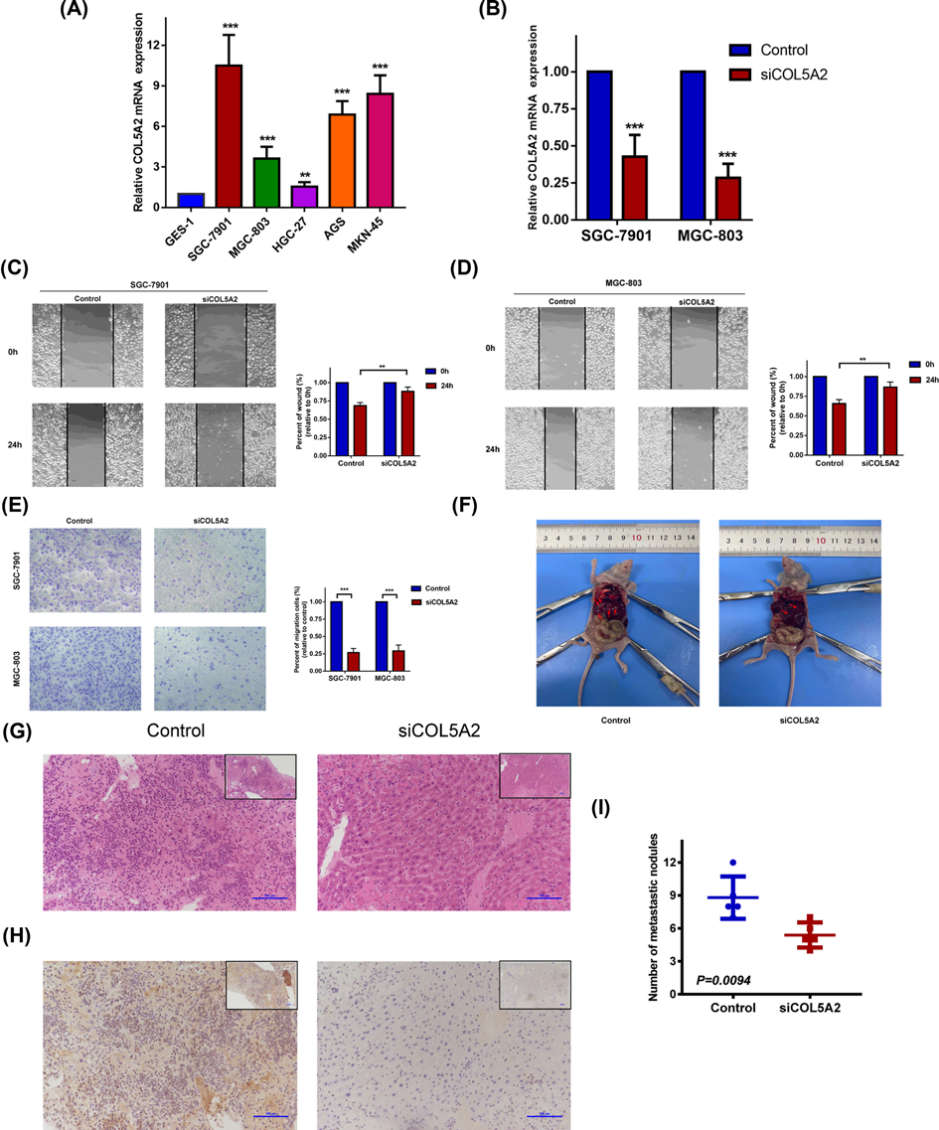
silenced COL5A2 decreased the migration of GC cells *in vitro* and *in vivo* (**A**) Comparison of COL5A2 expression in normal gastric epithelial cells (GES-1) and various gastric cancer cell lines (SGC-7901, MGC-803, HGC-27, AGS, and MKN-45). (**B**) COL5A2 expression in SGC-7901 and MGC-803 cells after interference. (**C** and **D**) Scratch wound detected the migration ability of gastric cancer cells comparing the control and siCOL5A2 groups. (**E**) Transwell detected the migration ability of gastric cancer cells comparing the control and siCOL5A2 groups. (**F**) Gross model of liver metastatic nodules in nude mice. (**G**) HE staining showed metastatic SGC-7901 cells. (**H**) Immunohistochemistry showed that COL5A2 in tissue was successfully knocked down in the siCOL5A2 group. (**I**) Statistical comparison of the number of metastatic nodules in the two groups. **P<0.01; ***P<0.001. *P*<0.05 represents statistical significance.

The mRNA expression levels of COL5A2 were compared in five GC cell lines and GES-1 (Figure 4A). SGC-7901 and MGC-803 cells were selected to further study. After siRNA transfection, the mRNA expression level of COL5A2 is shown in the Figure 4B. Scratch test showed that knockdown of COL5A2 could significantly reduce cell mobility (Figure 4C,D). Transwell assay showed that silencing COL5A2 could reduce the migration ability of cells (Figure 4E).

In order to verify the effect of COL5A2 on the migration of gastric cancer cells *in vivo*, SGC-7901 cells were injected into two groups of nude mice through tail vein. After one-week, physiological saline and siCOL5A2 were injected respectively. After three weeks, the number of metastatic nodules was calculated. It was found that the nude mice with siCOL5A2 had fewer and smaller metastatic nodules (Figure 4F–I).

### COL5A2 overexpression promoted the migration of GC cells *in vitro* and *in vivo*

**Figure 5:**
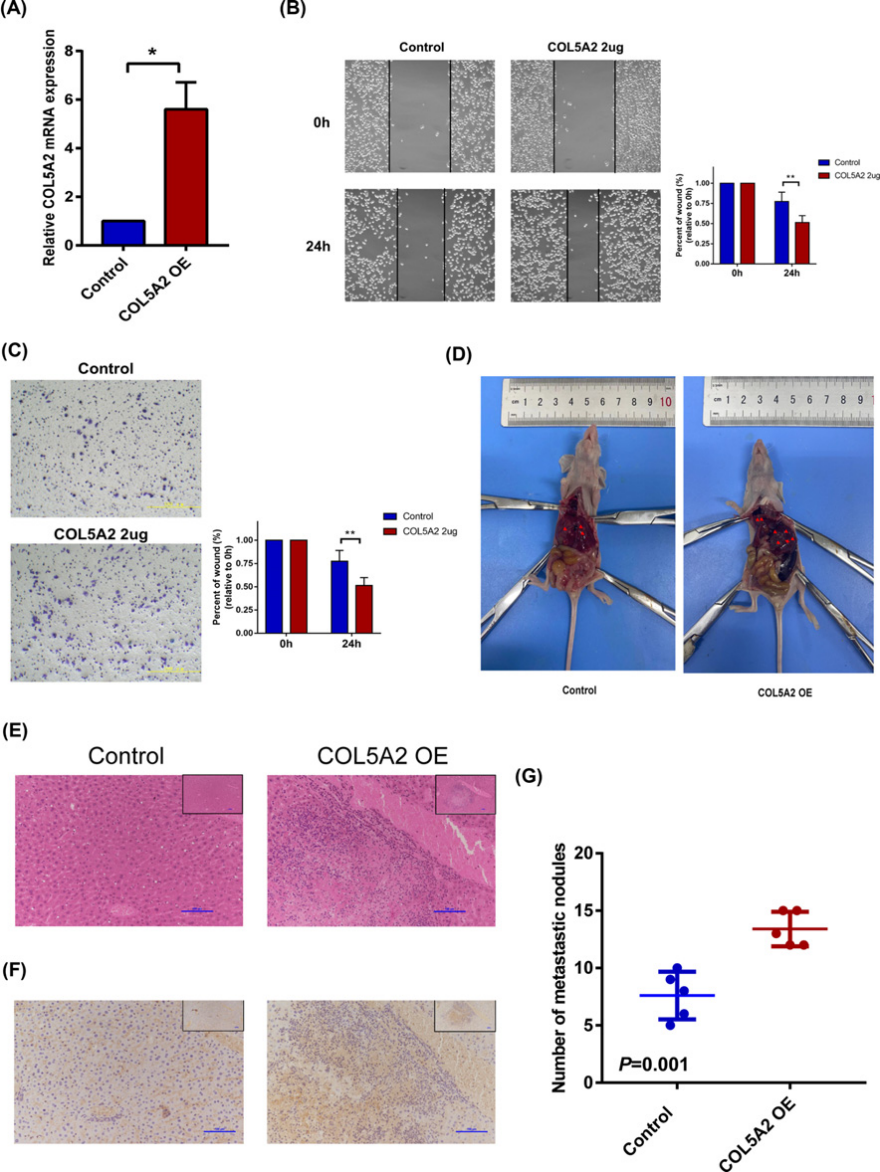
Overexpressed COL5A2 increased the migration of GC cells *in vitro* and *in vivo* (**A**) Overexpression efficiency of COL5A2 in HGC-27 cells. (**B**) Scratch wound detected the migration ability of gastric cancer cells comparing the control and COL5A2 OE groups. (**C**) Transwell detected the migration ability of gastric cancer cells comparing the control and COL5A2 OE groups. (**D**) Gross model of liver metastatic nodules in nude mice. (**E**) HE staining showed metastatic HGC-27 cells. (**F**) Immunohistochemistry showed that COL5A2 in tissue was successfully overexpressed in the COL5A2 OE group. (**G**) Statistical comparison of the number of metastatic nodules in the two groups. *P<0.05; **P<0.01. *P*<0.05 represents statistical significance.

The overexpression efficiency of HGC-27 cells was detected (Figure 5A). Scratch and migration experiments confirmed that overexpression of COL5A2 could promote the migration of gastric cancer cells, compared with the control group (Figure 5B,C). Similarly, *in vivo* experiment of tail vein injection of nude mice, the number of metastatic nodules in the COL5A2 OE group was significantly more than that in the control group, which further confirmed our conclusion (Figure 5D–G).

### Weighted co-expression network construction and module identification

**Figure 6:**
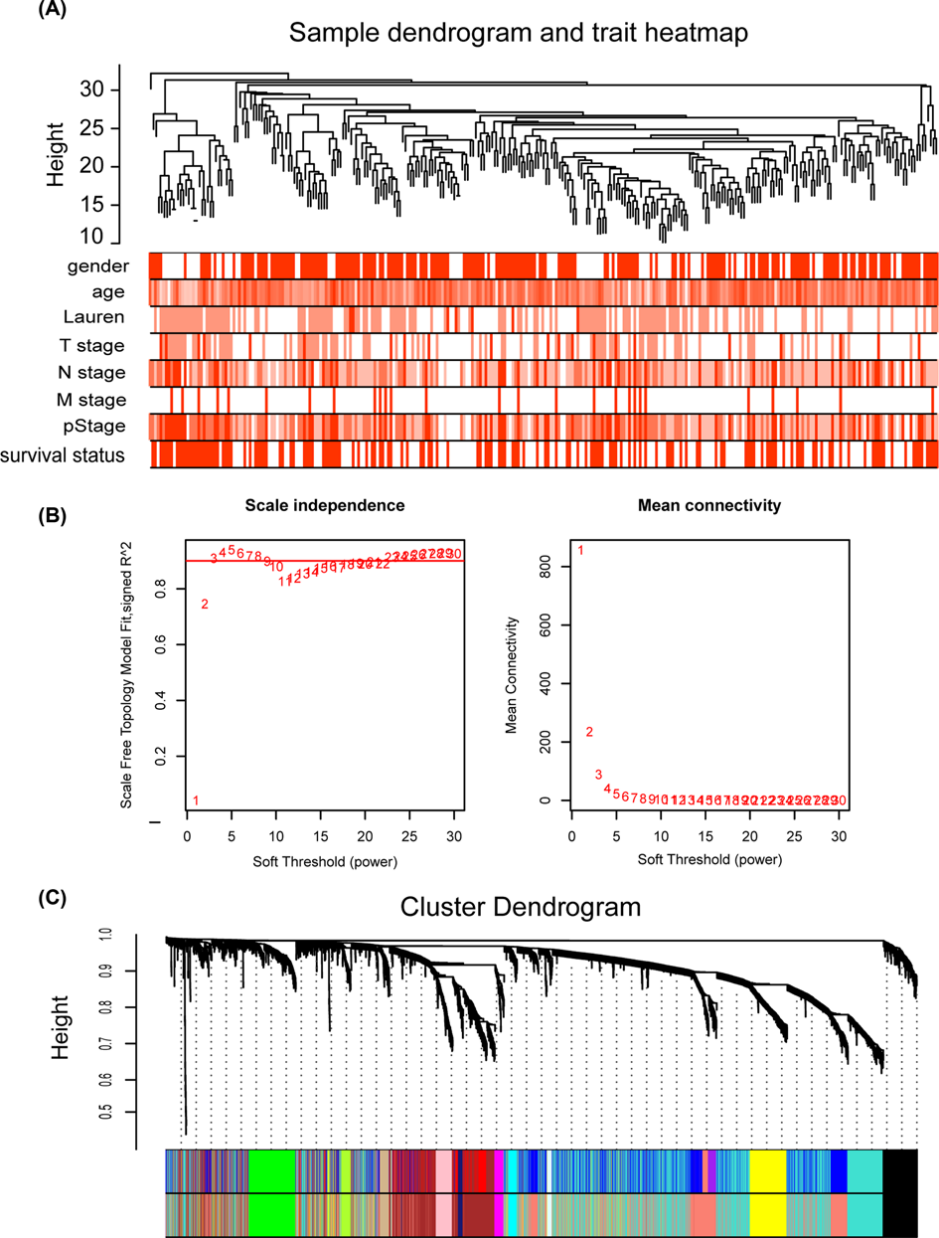
Construction of the weighted gene co-expression network (**A**) Clustering dendrogram of the clinical traits showed no obvious exception value. (**B**) Network topology analysis showed that when *β* = 3, the network topology met the threshold of 0.9 scale-free topology. The scale-free fit index in the left panel represents the soft-threshold power, and the mean connectivity in the right panels represents the soft-threshold power. (**C**) Clustering dendrogram of genes based on topological overlap. Each module served as a cluster of co-associated genes and was assigned a unique color.

After quality evaluation and data preprocessing, an expression matrix was formed from the 298 GC samples of the GSE62229 dataset. The clinical traits were shown in the heatmap of the clustering dendrogram (Figure 6A). With the variance in the top 25%, 5407 genes were screened out and used for subsequent co-expression analysis. When choosing the soft threshold, we calculated the network topology with power values from 1 to 20. As shown in Figure 6B, the power value of 3, which was the lowest power of the scale-free topological fit index of 0.9, was pitched on. Additionally, the mean connectivity met the scale-free network distribution at the power value of 3. After merging similar clusters, 13 different modules were identified that contained groups of genes with similar connection strengths (Figure 6C).

**Figure 7:**
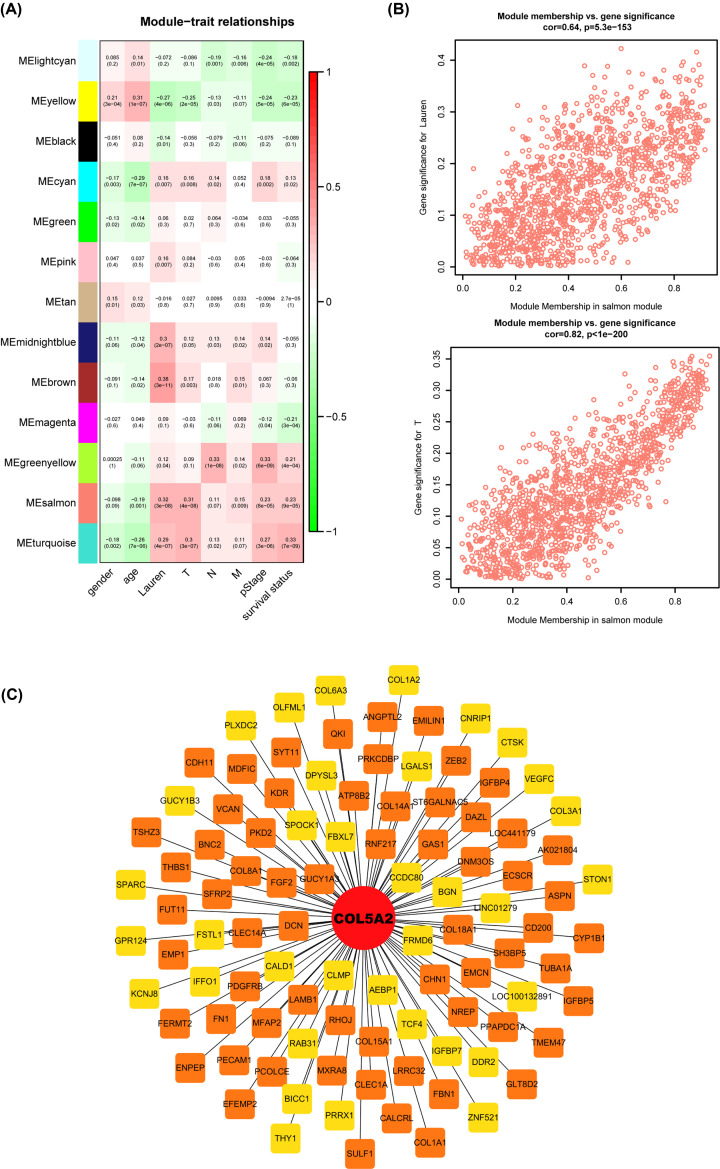
Analysis of the weighted gene co-expression network (**A**) Heatmap of the correlation and significant differences between the gene modules and clinical traits of GC. The correlation is displayed in the rectangle, while significant differences are shown in parentheses. (**B**) Two scatterplots of Gene Significance (GS) for the Lauren type (upper) and T stage (lower) versus Module Membership (MM) in the salmon module. (**C**) Protein–protein interactions (PPI) network of COL5A2 in the salmon module.

Finally, we found that COL5A2 was enriched into the salmon module (Figure 7A). and was highly correlated with T stage and Lauren stage (Figure 7B, *r* = 0.32, *P*=3e-8 and *r* = 0.31, *P*=4e-8). Interestingly, the salmon module was also found to be related to pStage (*r* = 0.23, *P*=8e-5) and survival status (*r* = 0.23, *P*=9e-5). Additionally, we selected the top 100 genes related to COL5A2 and constructed a visualized network using Cytoscope software (Figure 7C).

### Functional annotation and GSEA in the GSE62229 dataset and TCGA database

**Figure 8:**
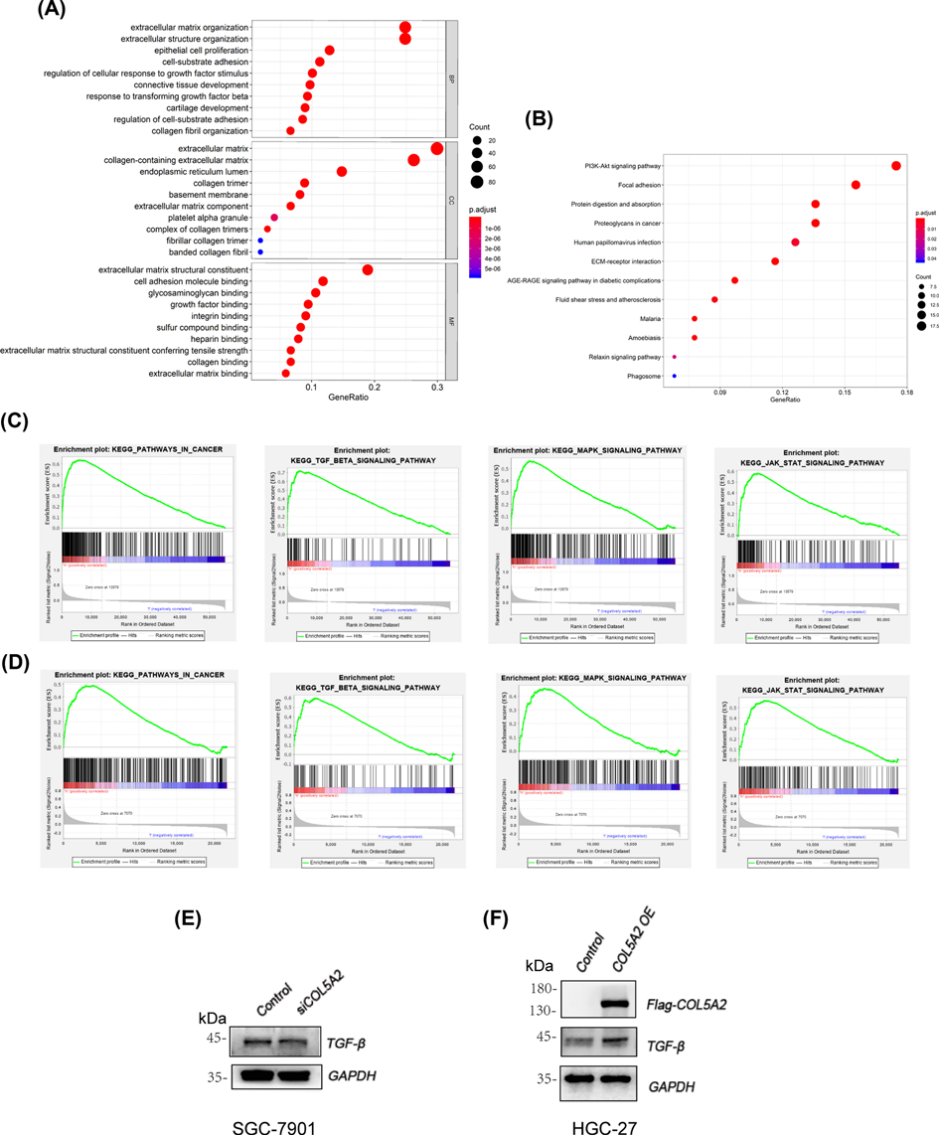
Functional annotation for COL5A2 in the salmon module (**A**) Enriched biological processes (BP), cellular components (CC,) and molecular functions (MF) of the salmon module. The *x*-axis displays the ratio of the total genes, and the *y*-axis displays the GO terms. (**B**) Enriched KEGG pathways of the salmon module. (**C**) GSEA of COL5A2 using the TCGA database. (**D**) GSEA of COL5A2 using the GSE62229 dataset. (**E**) The expression of TGF-β in control/siCOL5A2 SGC-7901 cells. (**F**) The expression of TGF-β in control/COL5A2 OE HGC-27 cells.

To understand the biological correlation of COL5A2, GO enrichment and KEGG pathway analyses were carried out. The top GO terms are shown in Figure 8A. The most enriched GO terms were as follows: BP (biological process), such as the extracellular matrix and structure organization, epithelial cell proliferation, and cell–substrate adhesion, CC (cellular component) such as the extracellular matrix, endoplasmic reticulum lumen, collagen trimer, and basement membrane, and MF (molecular function) such as cell adhesion molecular binding, glycosaminoglycan binding, and growth factor binding. Additionally, these genes were mainly enriched in the PI3K-Akt signaling pathway and focal adhesion, suggesting that the tumor microenvironment plays an important role in metastasis development (Figure 8B).

We performed GSEA of the GSE62229 dataset and TCGA database which revealed that COL5A2 was enriched in focal adhesion, ECM receptor interaction and regulation of actin cytoskeleton (Supplementary Figure S1). The GSEA results also showed that metastasis samples were significantly enriched in several well-known cancer-related pathways, such as the TGF-β, MAPK, and JAK2 signaling pathways (Figure 8C,D). The results provide clues into the in-depth mechanism of metastasis development. Western blot showed that the expression of TGF-β was decreased in silencing COL5A2, and overexpression of COL5A2 increased the expression of TGF-β (Figure 8E,F).

## Discussion

GC is a biologically and pathologically heterogeneous disease [23]. The prognosis of advanced GC has shown little improvement, and it is necessary to identify efficient prognostic biomarkers and therapeutic targets. In the present study, we first focused on the COL5 family, and chose COL5A2 as our target, according to the expression, OS and PFS data of the TCGA database. The analyses showed that COL5A2 was associated with T stage and Lauren stage and is involved in cancer-related pathways.

The expression level of COL5A2 is increased in various types of cancers, such as pancreatic cancer and colon cancer [9,24]. The up-regulation of COL5A2 is correlated with a poor prognosis in tongue cancer [8], a finding that was consistent with ours. Moreover, higher COL5A2 expression was associated with the Borrmann type, histological type, and T stage in the GC samples of our department, suggesting that COL5A2 might be a potential biomarker for GC tumorigenesis and progression.

WGCNA is a method that can highlight functional co-expression gene modules, and plays an important role in determining the potential mechanisms of malignancies, including breast cancer and colon cancer [16,25]. One main advantage of our study was that the WGCNA model of GSE62229 was constructed identify the module of COL5A2, and further explore the role of COL5A2 in GC. Eventually, we found that COL5A2 was enriched in the salmon module and was associated with T stage and Lauren stage, findings that are consistent with our IHC data. However, our study possessed the limitation of a small sample size and more databases need to be incorporated into future research.

Disorders of functions and cancer-related pathways are common in cancers [26,27]. Regarding GO and KEGG enrichment analyses, COL5A2 was involved in the extracellular matrix, focal adhesion, and PI3K-Akt signaling pathway. During cancer cell migration, Paluch et al. [28] proposed that adhesion to the matrix through a specific site is an essential step. Additionally, the PI3K-Akt signaling pathway plays an important role in cell migration, angiogenesis, and survival in GC [29,30]. In GSEA enrichment, cancer-related pathways, such as the TGF-β, MAPK, and JAK2 signaling pathways, were significantly identified. Notably, our previous study showed that TGF-β was an independent factor of the peritoneal metastasis of GC [31]. These results reveal the deeper mechanism of COL5A2 in the metastasis development of GC.

In conclusion, we aimed to select a COL5 family member with expression and survival significance and identified its potential molecular mechanism in advanced GC using bioinformatics analyses and clinical samples. Eventually, we used the TCGA database to select COL5A2 as our research target. WGCNA showed that COL5A2 was enriched in the salmon module, which was connected with the T stage and Lauren stage. Functional annotation demonstrated COL5A2 might be involved in the formation of the extracellular matrix, focal adhesion, and some cancer-related pathways. However, because the present study is mainly based on the analysis of open available datasets and clinical samples, further detailed experimental studies are needed to confirm the results in the future.

## Supplementary Material

Supplementary Figure S1

## Data Availability

All data in this article were available, and could find and use in the websites. K-M plotter (http://kmplot.com/), GEPIA (http://gepia.cancer-pku.cn/), and GSE62229 and GSE15459 (https://www.ncbi.nlm.nih.gov/gds).

## Abbreviations

DEG: different expression gene
GC: gastric cancer
GSEA: Gene Set Enrichment Analysis
RT-qPCR: real-time quantitative PCR
