# Supplementary material for: High expression of COL5A2, a member of COL5 family, indicates the poor survival and facilitates cell migration in gastric cancer
Source: Biosci Rep. 2021 Apr 9;41(4):BSR20204293. doi: 10.1042/BSR20204293 (PMC8039095; doi:10.1042/BSR20204293)

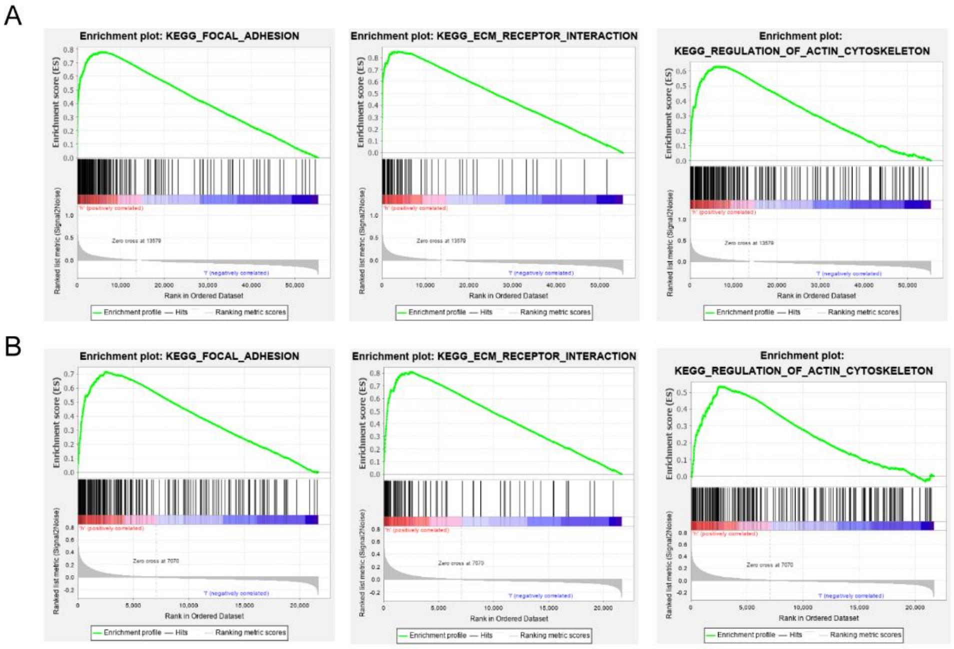

Supplement figure 1. Gene set enrichment analysis (GSEA) results of invasionand migration. (A) GSEA of COL5A2 using the TCGA database. (B) GSEA of COL5A2 using the GSE62229 dataset.

SGC-7901 TGF-β:

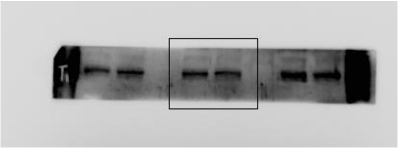

SGC-7901GAPDH:

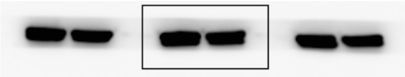

HGC-27 Flag-COL5A2

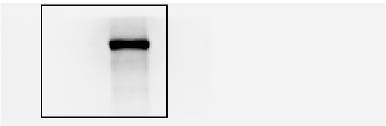

HGC-27 TGF-β：

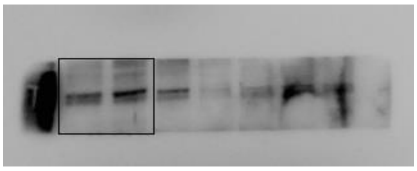

HGC-27 GAPDH:

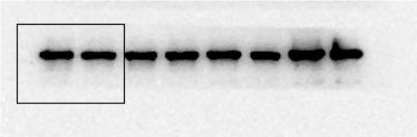

Supplement: Supplementary Figure S1 [file BSR-2020-4293_supp.pdf]
